# A Risk-Scoring Model Based on Evaluation of Ferroptosis-Related Genes in Osteosarcoma

**DOI:** 10.1155/2022/4221756

**Published:** 2022-03-28

**Authors:** Mingyang Jiang, Zifan Wang, Xiaoyu He, Yang Hu, Mingjing Xie, Yiji Jike, Zhandong Bo, Wentao Qin

**Affiliations:** ^1^Department of Bone and Joint Surgery, First Affiliated Hospital of Guangxi Medical University, Nanning 530021, China; ^2^Department of Burn Plastic and Wound Repair, The Affiliated Hospital of Youjiang Medical University for Nationalities, Baise 533000, China; ^3^Department of Thoracic Surgery, The Shenzhen Bao'an District Songgang People's Hospital, Shenzhen, China

## Abstract

**Background:**

Osteosarcoma (OS) is a bone malignancy frequently seen in pediatrics and has high mortality and incidence. Ferroptosis is an important cell death process in regulating the apoptosis and invasion of tumor cells, so constructing the risk-scoring model based on OS ferroptosis-related genes (FRGs) will benefit the evaluation of both treatment and prognosis.

**Methods:**

The OS dataset was screened from the Therapeutically Applicable Research to Generate Effective Treatments (TARGET) database, and OS-related FRGs were found through the Ferroptosis Database (FerrDb) using a multivariate Cox regression model, followed by the generation of the risk scores and a risk-scoring prediction model. Further systematical exploration for immune cell infiltration and assessing the prediction of response to targeted drugs was conducted.

**Results:**

Based on OS-related FRGs, a risk-scoring model of FRGs in OS was constructed. The six FRGs played a role in the carbon metabolism, glutathione metabolism, and pentose phosphate pathways. Results from targeted drug sensitivity analyses were concordant to pathway analyses. The response to targeted drugs statistically differed between the two groups with different risks, and the high-risk group presented a high sensitivity to targeted drugs.

**Conclusions:**

We identified a 6-ferroptosis-gene-based prognostic signature in OS and created and verified a risk-scoring model to predict the prognosis of OS at 1, 3, and 5 years for OS patients independently.

## 1. Introduction

Osteosarcoma (OS) is one of the malignancies frequently seen in pediatrics with high disability rates and mortality [1]. The 5-year survival rate of OS patients is improved to 50–60% with developed understanding of cancer pathogenesis and the updating of diagnostic methods [2]. The understanding of tumor biology has advanced considerably over the past decades [3]. As one of the vital cell death processes participating in the pathophysiology of cells, ferroptosis is involved in regulating apoptosis, invasion, and metastasis of tumor cells [4]. As a new programmed cell death, ferroptosis is iron-dependent and in contrast to apoptosis, cell necrosis, and autophagy. The primary mechanism is that unsaturated fatty acids from the cell membrane are catalyzed in the presence of divalent iron or ester oxygenase, which activates lipid peroxidation and induces cell death.

With the exploration of ferroptosis, plenty of evidence may hold out molecularly targeted therapies for OS patients. A previous study indicated that the mitogenic actions on osteoblasts were related to stimulation of G6PD activity [5]. Marinkovic et al. demonstrated that the correlation of p63 with G6PD and PGD predicts a poor prognosis using bioinformatics [6]. The other four FRGs were not subject to OS-related evidence, but they also played essential roles in other tumor pathways. For example, ACSF2 could be one of the FRGs to predict breast cancer [7]. Similarly, FADS2 was proved as a predicting FRG of bladder cancer.

As yet, the traditional approaches based on histopathologic diagnosis and tumor staging system for prognostic prediction of OS patients are not sufficient for precisely evaluating the outcomes [8]. It forces the development of robust and accurate prognostic biomarkers to assist clinicians to optimize therapy strategies. Hence, establishing an effective risk-scoring model based on FRGs in OS could assist in evaluating therapy and prognosis.

There are many online databases now, but there are few samples in the data set about OS. As a database for pediatric tumors, the TARGET database utilizes an integrative genomic approach to determine the molecular alterations during the onset and development of pediatric tumors and is aimed at using data to help guide the development of more effective and less toxic therapies [9]. And through data analysis, it generates useful drug targets and prognostic markers for researchers to develop new and more effective treatment options [10].

In this study, an OS dataset from TARGET was downloaded for the prediction of the OS occurrence based on ferroptosis. FRGs were screened and normalized. Then, a risk-scoring prediction model was constructed through the multiple COX regression model [11], and the Kyoto Encyclopedia of Genes and Genomes (KEGG) and gene ontology (GO) database were utilized to determine related biological process enrichment signaling pathways of FRGs.

## 2. Methods

### 2.1. Data Preparation

The gene set information including 85 samples (TARGET-OS) was contained as a training set derived from the TARGET database [11]. The patient information consisted of survival time and status, sex, age, disease at diagnosis, primary tumor site, specific tumor region, and eventual surgery (Table 1). The Ferroptosis Database (FerrDb) was utilized for the ferroptosis information collection of FRG selection [12].

### 2.2. Model Establishment

To establish our model, we combined univariate Cox-LASSO–multivariate Cox regression with the clinical factors and finally constructed the risk-scoring model using the selected FRGs. Univariate and multivariate Cox regression analyses were performed using R's “survival” package, and *P* < 0.01 was used as the filtering condition of univariate Cox [13]. To prevent large variance, we performed LASSO regression analysis using R's “GLMNET” package and determined *K* value by minimum lambda [11]. The gene at the minimum of the Akaike information criterion (AIC) was calculated and used as the variable to be included in the model, and each patient's gene expression level was used to evaluate the risk score, with the algorithm according to the previous studies [14, 15]. The median risk score of each patient is considered the reference standard for classifying the high and low groups, followed by the analysis of the survival of the two groups and drawing the survival curves using the Kaplan-Meier method (K-M method) [16]. In virtue of the critical parameters and model scores and in combination with various clinical factors, we drew a clinically relevant nomogram to predict the 1-, 3-, and 5-year survival, and the scales on nomograms represented the numerical ranges of each variable [17].

### 2.3. Model Validation

We used the package of “Survival ROC” to draw receiver operating characteristic curves (ROC) and “RMS” for the calibration to evaluate the accuracy of the predicted survival rates and ROC curves for the validation of each grouping variable [18].

### 2.4. GO and KEGG Analysis

We investigated the cellular components (CC), BP, and molecular function (MF) in the FRGs from the GO database. Furthermore, screened FRGs were analyzed for the functional pathway analysis of KEGG and for the functional enrichment analysis using R software and ClusterProfiler package [19]. We then used the “corrplot” package to analyze the relationships between FRGs by Pearson's correlation coefficient.

### 2.5. Gene Set Enrichment Analysis (GSEA)

GSEA is implemented to enrich gene sets and determine the distribution differences between whole gene sets and phenotypes, thereby achieving enrichment. The grouping file of the FRG expression differences and the downloaded expression matrix file of OS common transcription group were input into GSEA4.0.3 software [20]. The data sets used for enrichment were C2 and C5 molecular sets from the Molecular Characteristic Database (MSigDB), and the output results were adjusted to 100 sheets [21]. Finally, the enrichment gene sets were screened according to Normalized Enrichment Score (NES) > 1, False‐Discovery Rate (FDR) < 0.25, and *P* < 0.05.

### 2.6. Immune Cell Infiltration

The visualization was conducted for proportions of immune cell signatures in the training set. The cell infiltration level and the stromal content for OS samples were collected through the single-sample GSEA (ssGSEA) algorithm, and consensus clustering through the “ConsensuClusterPlus” package.

### 2.7. Prediction of Response to Targeted Therapy

Half-maximal inhibitory concentrations (IC_50_) of targeted therapeutic drugs were plotted using R's “ggplot2” and “pRRophetic” packages. Box plots represented the connection of the IC_50_s to two risk groups.

## 3. Results

### 3.1. Collation of FRGs

**Figure 1:**
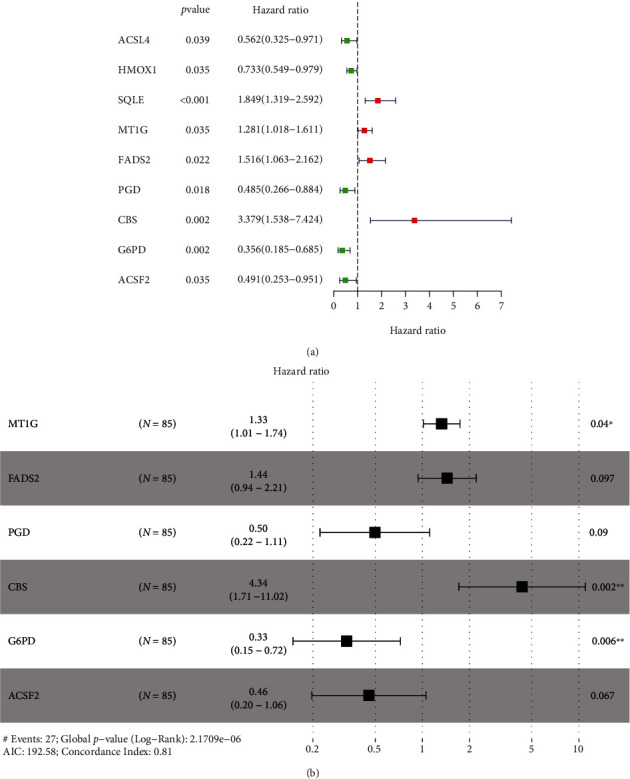
Development of prognostic ferroptosis-associated gene signature. (a) Forrest plot of univariate Cox regression. (b) Forrest plot of multivariate Cox regression.

Combining gene expression information of 85 OS patients obtained from TARGET and 61 FRGs on FerrDb, the OS-related FRGs as well as the expression information and clinical information were found. Nine survival-related FRGs were obtained as shown in Figure 1(a). We then used multivariate Cox regression and LASSO method to generate a categorizer to forecast OS according to the expression of FRGs. Finally, a combination of six genes (*ACSF2*, *CBS*, *FADS2*, *G6PD*, *MT1G*, and *PGD*) remained as predictors in the model (Figure 1(b)).

### 3.2. Data Preprocessing and Risk-Scoring Model Establishment

**Figure 2:**
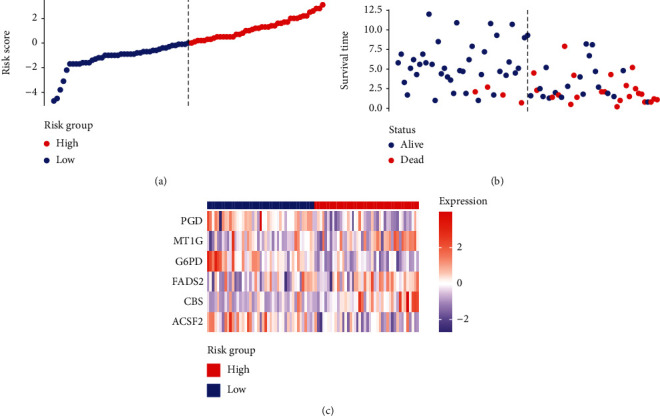
Establishment and validation of prognostic ferroptosis-associated gene signature. (a) Risk score plot, (b) survival status scatter plot, and (c) heatmap for the levels of *ACSF2*, *CBS*, *FADS2*, *G6PD*, *MT1G*, and *PGD*.

**Figure 3:**
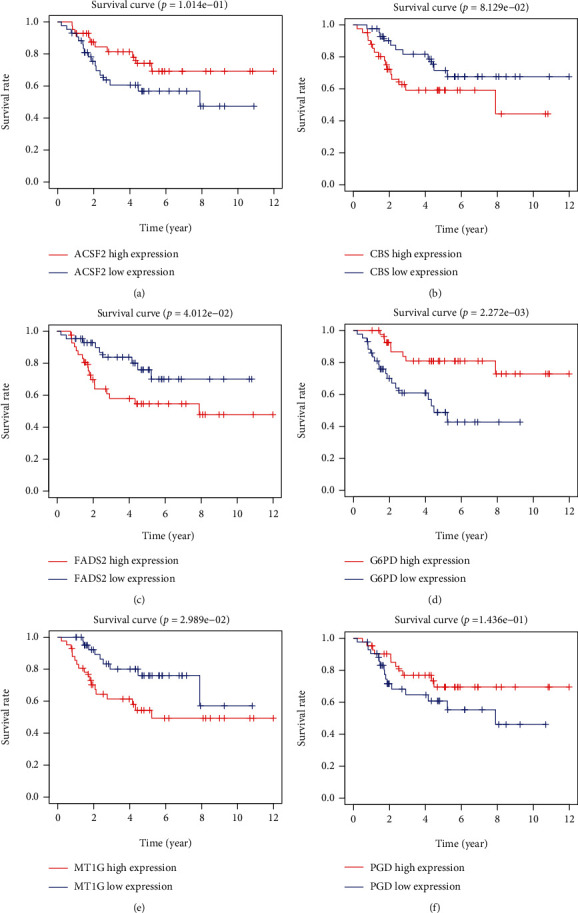
Survival rates of *ACSF2*, *CBS*, *FADS2*, *G6PD*, *MT1G*, and *PGD*.

**Figure 4:**
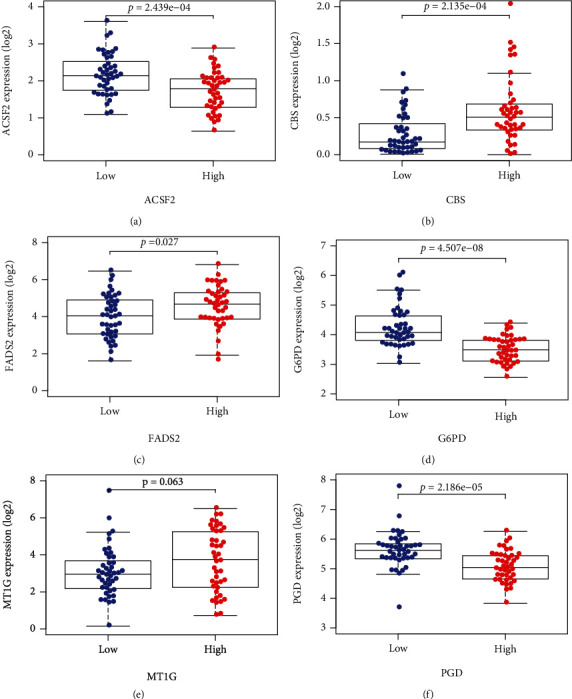
Expressions of *ACSF2*, *CBS*, *FADS2*, *G6PD*, *MT1G*, and *PGD*.

Based on the median risk score in the training set, the patients were separated into two risk groups: the low and the high. Survival analysis between groups showed that the risk score negatively correlated to the prognosis in the OS patients (Figures 2(a) and 2(b)). A heatmap was drawn to display the six genes level from their signatures (Figure 2(c)), showing lower expression of *PGD*, *G6PD*, and *ACSF2* of the high-risk group, together with higher levels of *MT1G*, *FADS2*, and *CBS*. The survival rates and gene expression levels of each hub FRG are shown in Figures 3 and 4.

### 3.3. Nomogram Development and Verification

**Figure 5:**
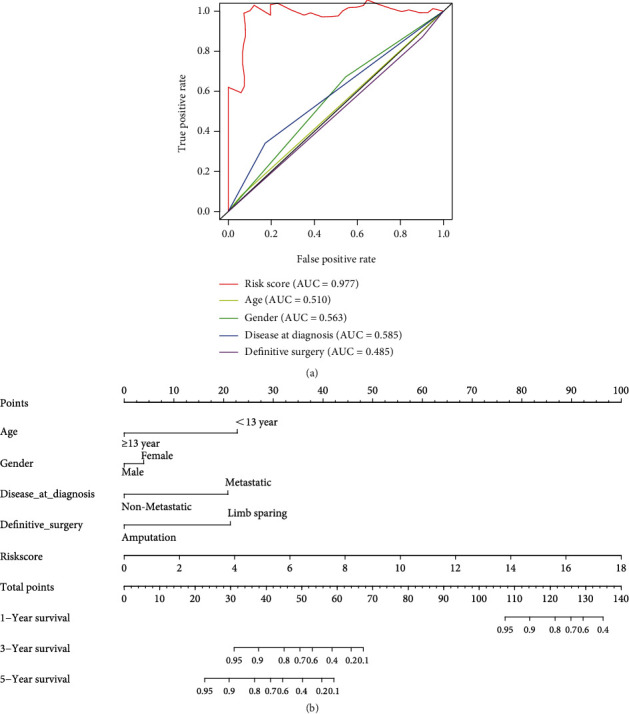
Construction of a predictive nomogram. (a) Independent related risk factors (sex, age, disease at diagnosis, definitive surgery, and risk score) were selected in the nomogram. (b) Nomogram for predicting 1-, 3-, and 5-year survival.

**Figure 6:**
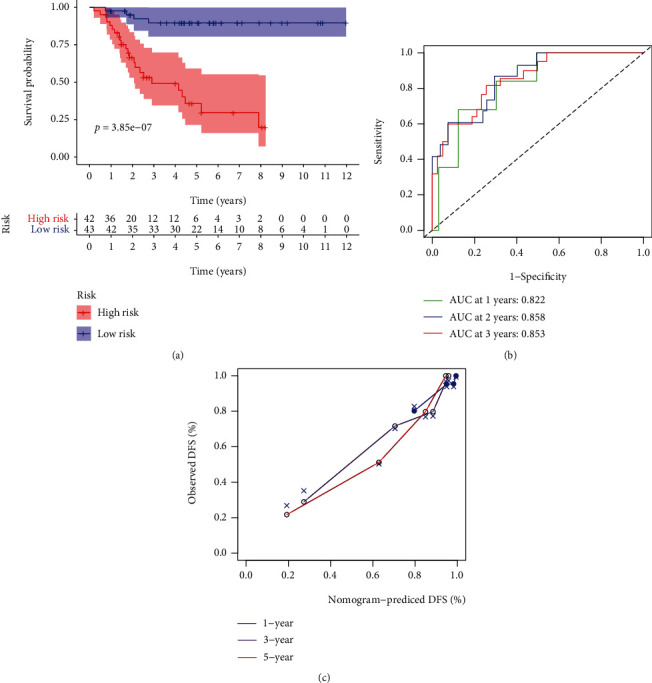
Validation of a predictive nomogram. (a) Kaplan-Meier curve comparing the survival rates, (b) ROC curve, and (c) calibration curve to judge the accuracy of the nomogram.

As shown in Figure 5, an OS risk estimation nomogram was formed combining the risk score and five independently related risk factors, including sex, age, disease at diagnosis, definitive surgery, and risk score. K-M curves indicated that over time, the survival rate of the low-risk group was higher than that of the high-risk group (Figure 6(a)). The prediction accuracy was evaluated in *C*-index (0.822) and calibration curve (Figures 6(b) and 6(c)).

### 3.4. KEGG and GO Analysis

**Figure 7:**
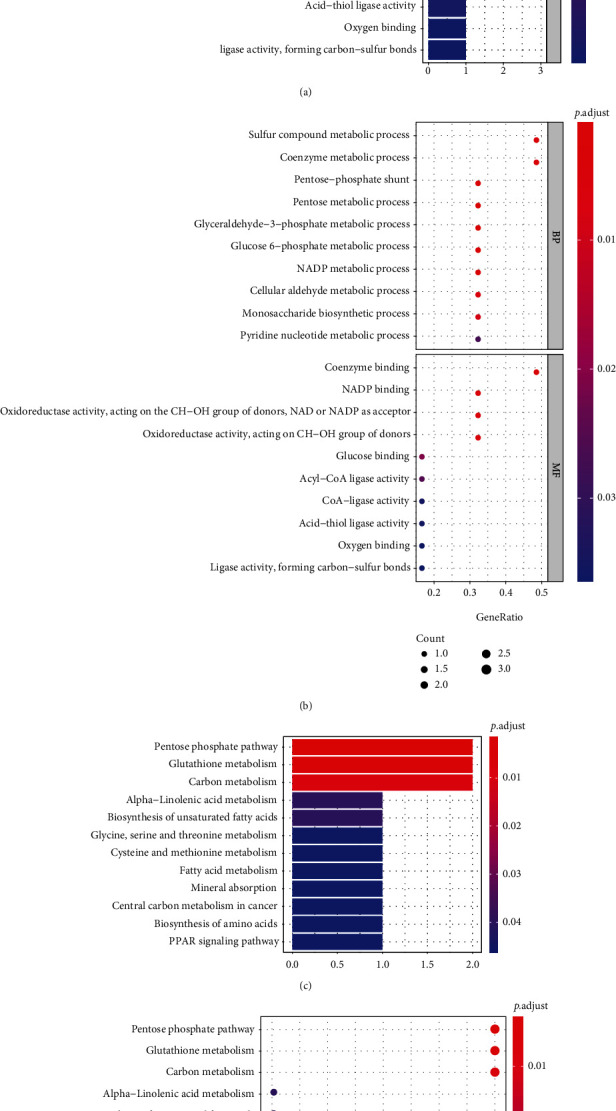
Significantly enriched GO annotations and KEGG pathways. (a) Bar plot and (b) bubble plot of gene ontology (GO) enrichment pathway. (c) Bar plot and (d) bubble plot of KEGG enrichment pathway.

The KEGG signaling pathway and GO functional process analysis were carried out specifying the biology pathways and processes associated with the six FRGs. The results indicated that these FRGs were functional in ferroptosis-related processes such as carbon metabolism, glutathione metabolism, and pentose phosphate pathway (Figure 7).

### 3.5. GSEA

**Figure 8:**
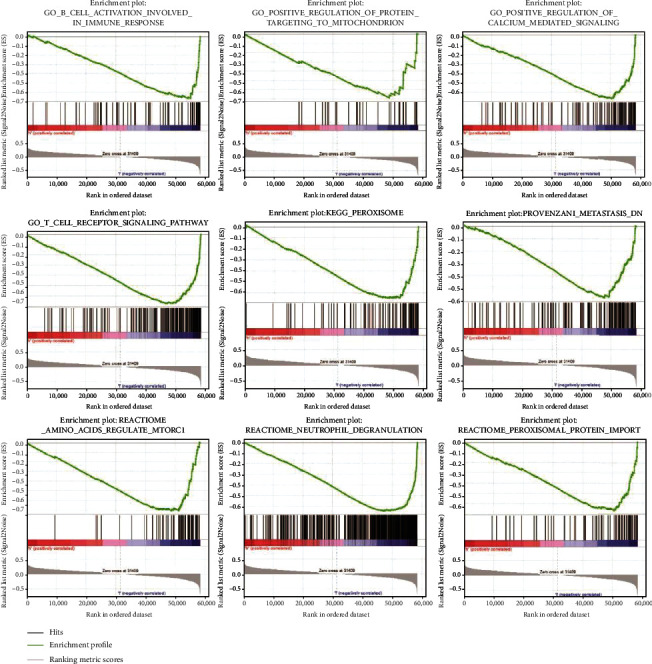
Gene set enrichment analysis results for *ACSF2*, *CBS*, *FADS2*, *G6PD*, *MT1G*, and *PGD*.

The stratification was conducted in OS patients depending on the median risk scores. The results disclosed the enrichment of six FRGs in the provenzani metastasis, peroxisomal protein import, neutrophil degranulation, amino acids regulating mTORC1, peroxisome, T cell receptor signaling pathway, regulation of calcium-mediated signaling, positive regulation of protein targeting to the mitochondrion, and B cell activation involved in immune response (Figure 8).

### 3.6. Immune Infiltrating

**Figure 9:**
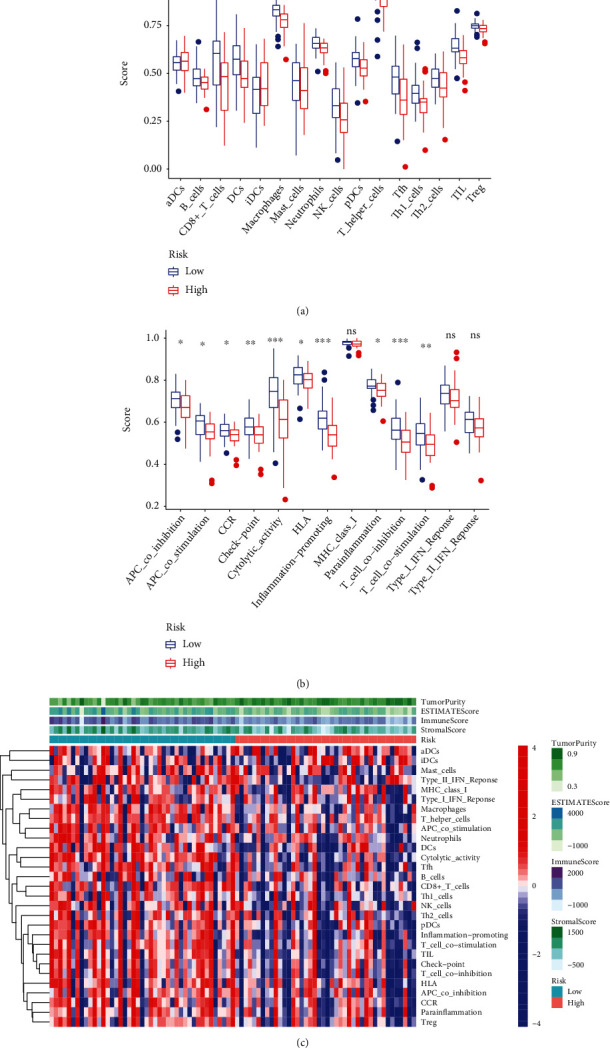
Tumor immune cell infiltration. (a) The 16 levels of immune cell infiltration. (b) The immune function. (c) Heatmap showing the infiltration level of immune signatures in different groups.

Except for activated dendritic cells (aDCs) and immature dendritic cells (iDCs), the numbers of other immune cells and immune functions of the low-risk group were significantly eminent compared to that of the high-risk group (Figures 9(a)–9(c)).

### 3.7. Response to Targeted Therapy

**Figure 10:**
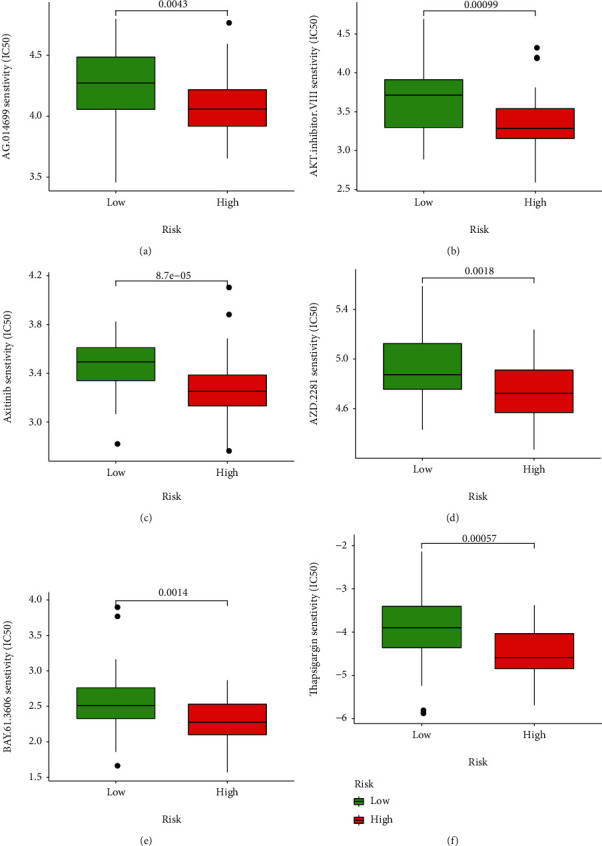
Prediction of response to targeted drugs. (a) AG.014699. (b) AKT.inhibitor.VIII. (c) Axitinib. (d) AZD.2281. (e) BAY.61.3606. (f) Thapsigargin.

Based on the predicted IC_50_s, the response to various targeted drugs differed significantly between the two groups with different risks. IC_50_s were lower in the high-risk group, indicating a higher sensitivity to targeted drugs (Figure 10).

## 4. Discussion

OS is the most common malignant tumor originated from mesenchymal tissue, which is prone to teenagers, recurrence, and lung metastasis with a poor prognosis. The main treatment of osteosarcoma is extensive or radical amputation, combined with chemotherapy. However, the multidrug resistance of osteosarcoma restricts its chemotherapy effect and long-term prognosis. Therefore, it is necessary to find new treatment methods.

Our research sifted six FRGs relating to OS that were possibly targeted for novel molecular therapy. The results from the risk prediction model in accordance with these six key FRGs showed noticeably separated survival curves between the two risk groups. In the time-dependent model, the risk score and death number have been elevated remarkably over time, suggesting the critical importance of these six FRGs on the prognosis prediction of OS. The key FRGs were good predictors of prognosis for OS patients, as shown by the ROC prediction results of 1-, 3-, and 5-year survival rates. Similarly, the six FRGs have shown promising outcomes in terms of clinical characterization studies.

According to the enrichment analyses results, OS's ferroptosis is closely related to carbon metabolism, glutathione metabolism, and the pentose phosphate pathway. Metabolic adaptation of cancer occurs as efficient cellular energy and biomass production alterations are indispensable for cancer onset and progression [22]. The catabolic and anabolic procedures in cancer metabolism can easily adjust to the elevated energy, and biological mass demands resulting from rapidly proliferating tumors. Malignant and metastatic cells of OS have elevated energy metabolism in comparison with the benign cells [23]. Among these metabolic pathways, the cysteine synthesis glutathione (GSH) pathway plays a leading role in the initiation of ferroptosis (Erastin induction pathway). Erastin is one of the small molecules found in chemical screening that can induce iron death in carcinogenic Ras mutant cell lines [24, 25]. In the process of iron death induced by Erastin, glutamate cysteine transporter, also known as X-C system, is the most important target of Erastin molecule. Cystine (the main form of intracellular cysteine) is mainly transferred into cells through glutamate cysteine transporter in the ratio of 1 : 1. Then, GSH and glutathione peroxidase 4 (GPX4) are synthesized in cells. GSH is mainly used as a cofactor in the process of protecting cells from oxidative damage, and GPX4 catalyzes the reduction of lipid peroxide to alcohols [26]. Therefore, targeting to cancer metabolism is ongoing to develop new therapies for cancer.

The risk-scoring model revealed the positive correction of risk scores with the sensitivity to targeted drugs. Prior investigations documented the effect of these drugs on cancer cytology. For example, axitinib is a potent and selective inhibitor of VEGFR-1–3. In transfected or endogenous RTK-expressing cells, axitinib potently blocked growth factor-stimulated phosphorylation of VEGFR-2 and VEGFR-3, thereby effectively inhibiting tumor growth, angiogenesis, and distant metastasis [27, 28]. Fritsche-Guenther et al. proved that AKT2 is a critical signal molecule in the insulin signaling pathway, which needs to induce glucose transport [29]. In our study, the group with high risk presented high sensitivity to AKT inhibitor VIII. Therefore, inhibiting AKT to interfere with glucose metabolism and then controlling the occurrence or development of ferroptosis can be considered the future research direction.

It has been reported that ferroptosis can improve the antitumor effect of immunotherapy by activating CD8+ T cells, but whether FRG affects the occurrence and development of OS by regulating the immune state of the tumor microenvironment is still unclear [30]. In the process of establishing and verifying the risk-scoring model, we found that OS patients with different FRG expression matrixes showed different immune states, and patients with more active immune states had better prognosis. The tumor-associated immune response vitally partakes in tumor cell infiltration, whereas ferroptosis critically regulates the tumor-related immune responses [31]. The immune cell infiltration analysis in this study indicated that the immune functions and the numbers of immune cells, except for aDCs and iDCs, are noticeably higher in the low-risk group, indicating the ferroptosis-related, antitumor immune response processes that reduce the risk of death in low-risk patients.

Some limitations exist in this study. For example, the sample size is comparatively insufficient, which needs future study to include more samples to evaluate the model performance further and elucidate the latent mechanism.

## 5. Conclusions

To sum up, a prognostic signature of OS based upon six FRGs was determined, and a risk-scoring model based on six OS-related FRGs was established. This risk-scoring model shows commendable performance to independently evaluate the prognosis of OS at 1, 3, and 5 years, which will provide the potential guidance of OS targeted therapy.

## Figures and Tables

**Table 1:** Clinicopathological characteristics of OS patients from TARGET database.

Characteristics		Patients (*N* = 85)	
		No.	%
Sex	Female	37	43.53
	Male	47	55.29
	Unknown	1	1.18
Age	≤14 (median)	44	51.76
	>14 (median)	40	47.06
	Unknown	1	1.18
Race	Race	51	60.00
	Asian	6	7.06
	Black or African American	7	8.24
	Unknown	21	24.71
Disease at diagnosis	Metastatic disease	21	24.71
	Nonmetastatic disease	63	74.12
	Unknown	1	1.18
Primary tumor site	Arm/hand	6	7.06
	Leg/foot	76	89.41
	Pelvis	2	2.35
	Unknown	1	1.18
Vital status	Dead	27	31.76
	Alive	58	68.24

## Data Availability

The data used to support the findings of this study are included within the article.
